# Inorganic phosphate modifies stationary phase fitness and metabolic pathways in *Lactiplantibacillus paraplantarum* CRL 1905

**DOI:** 10.3389/fmicb.2024.1343541

**Published:** 2024-02-27

**Authors:** Mario Araoz, Mariana Grillo-Puertas, Alejandra de Moreno de LeBlanc, Elvira María Hebert, Josefina María Villegas, Viviana Andrea Rapisarda

**Affiliations:** ^1^Instituto Superior de Investigaciones Biológicas (INSIBIO), CONICET-UNT, and Instituto de Química Biológica, “Dr. Bernabé Bloj”, Facultad de Bioquímica, Química y Farmacia, UNT, San Miguel de Tucumán, Argentina; ^2^Centro de Referencia para Lactobacilos (CERELA-CONICET), San Miguel de Tucumán, Argentina

**Keywords:** lactic acid bacteria, stationary phase, polyphosphate, proteomics, biofilm

## Abstract

Inorganic phosphate (Pi) concentration modulates polyphosphate (polyP) levels in diverse bacteria, affecting their physiology and survival. *Lactiplantibacillus paraplantarum* CRL 1905 is a lactic acid bacterium isolated from quinoa sourdough with biotechnological potential as starter, for initiating fermentation processes in food, and as antimicrobial-producing organism. The aim of this work was to evaluate the influence of the environmental Pi concentration on different physiological and molecular aspects of the CRL 1905 strain. Cells grown in a chemically defined medium containing high Pi (CDM + P) maintained elevated polyP levels up to late stationary phase and showed an enhanced bacterial survival and tolerance to oxidative stress. In Pi sufficiency condition (CDM-P), cells were ~ 25% longer than those grown in CDM + P, presented membrane vesicles and a ~ 3-fold higher capacity to form biofilm. Proteomic analysis indicated that proteins involved in the “carbohydrate transport and metabolism” and “energy production and conversion” categories were up-regulated in high Pi stationary phase cells, implying an active metabolism in this condition. On the other hand, stress-related chaperones and enzymes involved in cell surface modification were up-regulated in the CDM-P medium. Our results provide new insights to understand the CRL 1905 adaptations in response to differential Pi conditions. The adjustment of environmental Pi concentration constitutes a simple strategy to improve the cellular fitness of *L. paraplantarum* CRL 1905, which would benefit its potential as a microbial cell factory.

## 1 Introduction

Lactic acid bacteria (LAB) belong to an economically important group of Gram-positive microorganisms, whose main characteristic is the production of lactic acid from carbohydrate fermentation. Most technological applications expose these microorganisms to different stressful conditions (i.e., acidity, high temperature, and salt concentrations, among others), which could affect the physiological status of the bacterial cells (Mills et al., 2011; Papadimitriou et al., 2016). Furthermore, during passage through the gastrointestinal tract (GIT), bacteria must also endure the harsh conditions imposed (Mendonça et al., 2023). Therefore, the ability of LAB to withstand these hazardous conditions is crucial to ensure their benefits by maintaining the desired characteristics and viability (Tripathi and Giri, 2014; Zhang et al., 2018).

*Lactiplantibacillus paraplantarum* exhibits outstanding probiotic qualities, such as GIT tolerance, adhesion, antioxidant, and antibacterial properties. It also presents a potential to be used as starter in the production of a variety of fermented foods (Brown et al., 2017). In particular, *L. paraplantarum* CRL 1905, isolated from a laboratory quinoa sourdough preparation (Ruiz Rodríguez et al., 2016), presents relevant attributes as a promising agent for biotechnological approaches. This strain has versatile metabolism and functional applications, such as acidification activity, production of bioactive compounds and exopolysaccharides, and shows a high susceptibility to antibiotics of clinical importance as defined by the European Food Safety Authority (EFSA). It has been recently reported that CRL 1905 supernatant exhibited antifungal activity against the most common pathogens of citrus fruit, controlling lemons spoilage and extending their shelf life (Volentini et al., 2023). On the other hand, Teran et al. (2021) demonstrated that the intracellular extract of this strain could be used as a potential strategy to prevent or treat neurodegenerative diseases.

Polyphosphate (polyP) is a linear polymer formed by tens to hundreds of Pi linked by phosphoanhydride bonds (Kornberg, 1995). In bacteria, this polymer has been involved in virulence and biofilm formation, protein-like chaperone ability, and resistance to different environmental stress agents (metals, oxidants, acids, or UV radiation) (Kornberg et al., 1999; Grillo-Puertas et al., 2012, 2014, 2015; Nikel et al., 2013; Gray et al., 2014). Generally, polyP concentration in bacterial cells showed a sharp increase during the early logarithmic phase of growth, undergoing a gradual decrease to reach basal levels at the stationary phase (Nesmeyanova, 2000; Saiki et al., 2016). However, in several bacteria, polyP levels can be modulated by modifying inorganic phosphate (Pi) concentration in the culture media, leading to physiological adaptations that were correlated with variations of intracellular polymer levels during the stationary phase (Schurig-Briccio et al., 2009a and, 2009b; Grillo-Puertas et al., 2012, 2014, 2015, 2016, and, 2018). At present, studies comprising physiological and molecular responses related to different Pi concentrations in LAB are scarce (Alcántara et al., 2014; Correa Deza et al., 2017).

The aim of this study was to investigate the effects of Pi concentration on the different pathways of *L. paraplantarum* CRL 1905. Phenotypic and proteomic data showed that Pi concentration in the culture media affects bacterial fitness during the stationary phase, suggesting that Pi modulation could serve as a simple strategy to enhance the robustness of lactobacilli for potential technological applications.

## 2 Materials and methods

### 2.1 Bacterial strains, growth conditions, and media

*L. paraplantarum* CRL 1905 was originally isolated from Real Hornillos quinoa sourdough (Ruiz Rodríguez et al., 2016). This strain belongs to the public Culture Collection of the Centro de Referencia para Lactobacilos (CERELA-CONICET, Tucumán, Argentina). LAB cultures were propagated twice in MRS broth (Britania, Argentina) at 37°C for 16 h. Cells were harvested by centrifugation at 8,000 × g for 10 min and washed twice in sterile 0.85% (w/v) saline solution to eliminate residual nutrients. Washed cells were used to inoculate a chemically defined medium (CDM) (Hebert et al., 2004) to a final A_560nm_ of 0.1. CDM was modified in its original Pi content to achieve final concentrations of 2 or 60 mM potassium phosphate (named CDM-P or CDM + P, respectively). When indicated, media with different Pi concentrations were used. Cells were grown at 37°C under static conditions for different times.

### 2.2 Determination of polyP levels

Intracellular polyP levels were measured using a 4′, 6-diamidino-2-phenylindol (DAPI, Sigma, United States) based fluorescence approach (Aschar-Sobbi et al., 2008). Briefly, cells were washed twice with buffer T (100 mM Tris–HCl, pH 8) and resuspended in the same buffer. Measurements were performed at 37°C with constant agitation in cuvettes containing cell suspensions in buffer T at an A_560nm_ of 0.02 with 17 μM DAPI and 0.00075% SDS and chloroform for cell permeabilization. Then, the mixture was incubated for 10 min at 37°C with agitation. The DAPI fluorescence spectra (excitation, 415 nm; emission, 445–650 nm) were recorded using an ISS PCI spectrofluorometer (ISS Inc., Champaign, IL, United States). Fluorescence of DAPI-polyP complex at 550 nm was used as a measurement of intracellular polyP since emissions from free DAPI and DAPI-DNA are minimal at this wavelength.

### 2.3 Viability determination

The long-term survival of *L. paraplantarum* CRL 1905 was analyzed by culturing the cells statically at 37°C in CDM-P or CDM + P for 24, 48, and 120 h. Cell viability was quantitatively determined by plating the cells onto MRS-agar plates for 48 h at 37°C, and expressed as colony forming units per milliliter (CFU mL^−1^).

### 2.4 Flow cytometry analysis

The percentages of live, injured, and dead cells were determined by flow cytometry. 48 h- CRL 1905 cells grown in CDM-P or CDM + P were stained with a cell viability kit (BD™ Biosciences, United States). Cells were suspended in phosphate-buffered saline (PBS) to approximately a concentration of 10^6^ cells mL^−1^ and incubated at room temperature for 5 min with 420 μM for thiazole orange and 48 μM for propidium iodine. Samples were analyzed using a FACS Calibur flow cytometer (BD™Biosciences, United States) using two different wavelengths, 488 nm and 635 nm. Assays were carried out in duplicate, and data analysis was performed using FCS Express 4Flow Cytometer (*De Novo* Software, Glendale, CA, United States).

### 2.5 DPPH radical scavenging assay

The 1,1-diphenyl-2-picrylhydrazyl (DPPH) radical assay was performed to evaluate the radical scavenging activity of CRL 1905 cells grown in different media. 7 and 48 h-cells were harvested by centrifugation at 8,000 × *g* for 10 min at 4°C. The obtained pellets were washed twice and resuspended in saline solution (A_560nm_ = 1). 1.0 mL of ethanolic DPPH radical solution (0.2 mM) was added to 1.0 mL of suspensions of cells grown in CDM-P or CDM + P. The mixtures were incubated in the dark at 25°C for 30 min. Then, suspensions were centrifuged at 6000 × *g* for 10 min, and the A_517 nm_ of the obtained supernatants were measured. The DPPH radical scavenging ability was calculated as follows: scavenging activity (%) = [1 − (A_sample_ − A_blank_)/A_control_] × 100%, where A_sample_, A_blank_ and A_control_ represent the A_517 nm_ of the sample, blank and control, respectively. Controls used deionized water instead of intact cells, and blanks used anhydrous ethanol instead of DPPH.

### 2.6 Lipid peroxidation

Lipid peroxidation, normally used to assess oxidative damage, was determined by thiobarbituric acid reactive substances (TBARS) assay, as reported by Castro et al. (2021) with minor modifications. Briefly, suspensions of cells grown in CDM-P or CDM + P were centrifuged, washed twice with sterile saline solution, and lysed by adding glass beads to be vortex-agitated. Then, cells were centrifuged at 8,000 × *g* for 10 min at 4°C, and the supernatants were collected. 12.5 μL of 20% trichloroacetic acid (TCA) was added to 500 μL of supernatant to determine malondialdehyde (MDA). The reaction mixture contains 150 μL of cell extract, 150 μL of H_2_O, 100 μL 0.1 M EDTA, and 600 μL of 1% thiobarbituric acid in 0.05 M NaOH. A reaction blank containing H_2_O without cell extract was also prepared. The reaction mixture was incubated at 100°C for 15 min. The tubes were cooled and MDA was measured spectrophotometrically at 532 nm. TBARS content (expressed in pmol mg protein^−1^) was determined using a molar extinction coefficient 156 mM^−1^ cm^−1^. Protein concentration was determined by the Bradford method (Bio-Rad, United States).

### 2.7 Tolerance to hydrogen peroxide

Microbial survival under oxidative stress was assessed by exposing CRL 1905 strain to hydrogen peroxide. Cells grown in CDM + P or CDM-P for 48 h were centrifuged and washed twice, resuspended in physiological solution and exposed to 10 mM hydrogen peroxide at 37°C for 15 min. The survival rate was calculated as follows: Survival rate (%) = (log_CFU_ N_1_/log_CFU_ N_0_) x 100, where N_1_ is the total viable count after treatment with H_2_O_2_, and N_0_ is the total viable count before treatment (Guo et al., 2009).

### 2.8 Scanning electron microscopy (SEM)

For SEM analysis, *L. paraplantarum* CRL 1905 cells grown in CDM-P or CDM + P during 48 h were fixed with 2.5% glutaraldehyde, 2.5% paraformaldehyde, acetone, and ethanol dehydrated, and gold coated with anion sputter JFC-1100 (JEOL). Samples under study were then attached to aluminum holders and analyzed using a Carl Zeiss SUPRA-55 scanning electron microscope from CIME (Centro Integral de Microscopía Electrónica, CONICET, Tucumán, Argentina) with a resolution of 1.0 nm at 15 kV and 1.7 nm at 1 kV in high-vacuum (HV) mode and 2 nm at 30 kV in variable-pressure mode.

### 2.9 Quantification of biofilm formation

Biofilm formation was assayed based on the cells ability to adhere and grow on polystyrene microtiter plates and stained with crystal violet. Cells were grown in microtiter plates containing CDM-P or CDM + P under static conditions at 37°C for 48 h. Unattached cells were removed by washing the plates with deionized water. 0.1% crystal violet solution was added to each well. Plates were incubated at room temperature for 20 min and rinsed 3 times with water. The absorbed crystal violet was extracted with 95% ethanol and measured at A_595nm_ (Spectra MaxPlus384 Absorbance Microplate Reader, United States). Six replicates were performed for each experimental condition.

### 2.10 Confocal laser scanning microscopy (CLSM)

Biofilm formation of *L. paraplantarum* CRL 1905 cells was carried out in CDM-P or CDM + P medium, using 6-well polystyrene plates with a glass coverslip inside. After incubation for 48 h, unattached cells were removed, and coverslips were washed with PBS and dried at 37°C for 10 min. The adhered cells were immersed in PBS containing 1.5 μL of dye Syto9 and incubated for 20 min in the dark. The coverslips were then rinsed with sterile water and removed from the wells. Images were captured using a Zeiss Confocal Microscope LSM800 at the IMMCA (Instituto de Investigación en Medicina Molecular y Celular Aplicada, CONICET-UNT-SIPROSA, Tucumán, Argentina).

### 2.11 Label-free proteomics

Cells grown during 48 h in CDM-P or CDM + P media were harvested by centrifugation (8,000 × g, 10 min, 4°C), washed with 50 mM Tris–HCl buffer, and resuspended in 50 mM Tris–HCl buffer (pH 7.5) containing 1 mM PMSF and 10 mM EDTA. Cell extracts were obtained by homogenizing the bacterial suspensions with glass beads (0.15- to 0.25-mm diameter; Sigma, United States) in a MiniBeadbeater-16 Cell Disrupter (Biospec, United States) for 10 min. Glass beads, unbroken cells, and cell debris were removed by centrifugation (14,000 × g, 10 min, 4°C). Protein concentration was determined by the bicinchoninic acid assay (BCA assay). The label-free proteomics was conducted at the Proteomics Core Facility CEQUIBIEM (University of Buenos Aires-CONICET). 30 μg protein extract of each sample was reduced with 20 mM dithiothreitol (DTT), alkylated with 50 mM iodoacetamide for 45 min in the dark, and then digested with trypsin overnight. Peptides extraction was performed with acetonitrile. Samples were dried with a SpeedVac device, resuspended with 30 μL of 0.1% trifluoroacetic acid, and desalted with a ZipTip C18 column (Merck). Then, peptides were analyzed by nanoHPLC (EASY-nLC 1000) coupled to an Orbitrap technology mass spectrometer (Q-Exactive with High Collision Dissociation cell and Orbitrap analyzer). Sample ionization was carried out by nanoelectrospray (Thermo Scientific brand, model EASY-SPRAY. Spray voltage: 1.8 kV). The instrument was equipped with an HCD (High Collision Dissociation) cell and an Orbitrap analyzer yielding the identification of peptides simultaneously to their separation by chromatography. The analysis of the raw files delivered by the mass spectrometer was performed by using the Proteome Discover software (version 2.1.1.21 Thermo Scientific) using the peptide peak area for identification. Searches were performed throughout the *L. plantarum* (strain ATCC BAA-793/NCIMB8826/WCFS1) protein sequence database (https://www.uniprot.org/proteomes/UP000000432). The mass spectrometry proteomics data have been deposited to the ProteomeXchange Consortium via the PRIDE (Perez-Riverol et al., 2019) partner repository with the dataset identifier PXD018988. The statistical data analysis and visualization were performed using Perseus software (version 1.5.6.0, Max Planck Institute, Germany) (Tyanova et al., 2016). Proteins displaying at least 1.5-fold changes, with a *value of p* ≤0.05, were considered as differentially expressed. In some exceptional cases, proteins with *value of p* of <0.1 and fold change values of ≥2.5 were also considered as regulated due to their biological significance.

### 2.12 Statistical analysis

When indicated, data were subjected to analysis of variance (ANOVA) followed by Tukey’s test with Infostat for Windows, 2016 version (Di Rienzo et al., 2016).

## 3 Results

### 3.1 Pi concentration modulates polyP levels in *Lactiplantibacillus paraplantarum* CRL 1905 during the stationary phase

**Figure 1:**
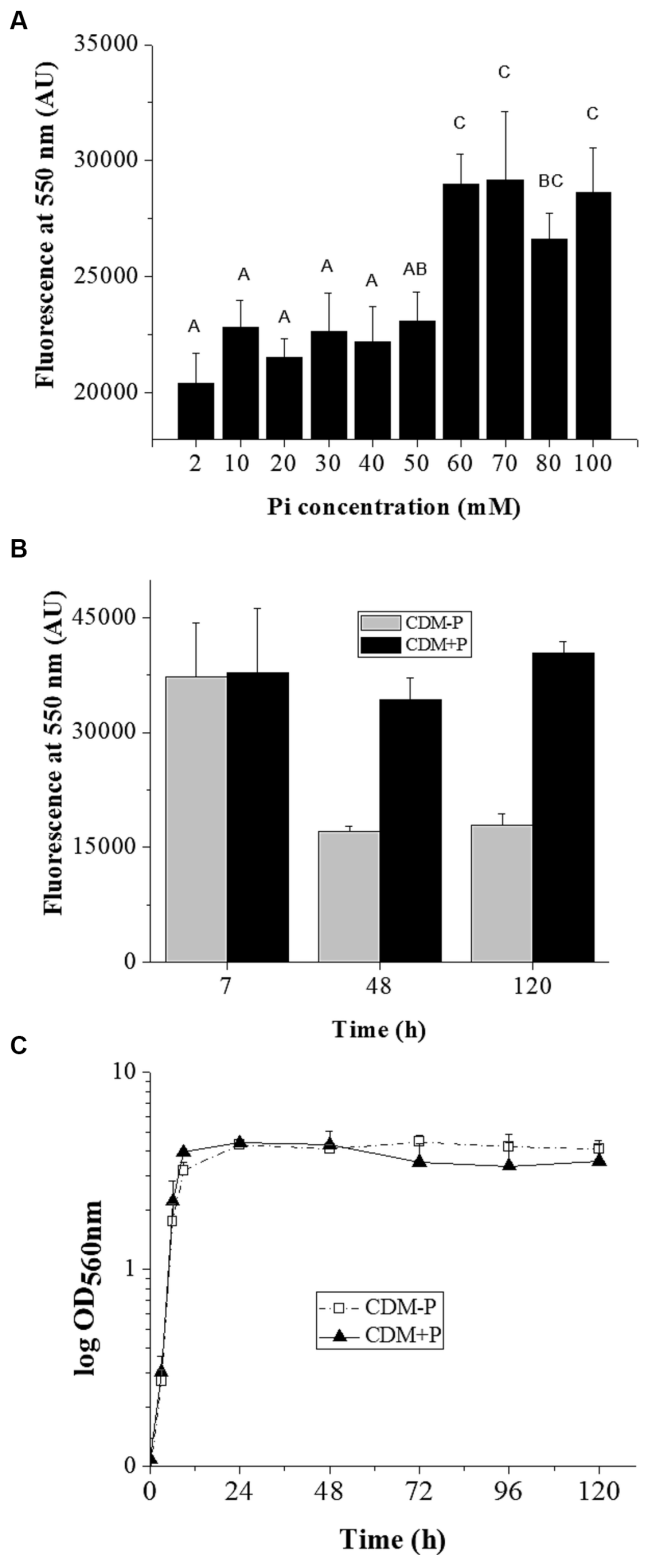
Influence of medium Pi concentration on intracellular polyP levels and bacterial growth. **(A)** Fluorescence emission at 550 nm of the DAPI- polyP complex measured in 48 h- CRL 1905 cells grown in CDM supplemented with the indicated Pi concentrations. **(B)** Fluorescence of the DAPI- polyP complex determined in *Lactiplantibacillus paraplantarum* CRL 1905 grown in CDM supplemented with 2 (CDM-P) or 60 (CDM + P) mM Pi at 7, 48 and 120 h. **(C)** Representative growth curves of CRL 1905 strain in the indicated media measured by absorbance at 560 nm during 120 h. Fluorescence is expressed in arbitrary units (AU) and the values are the average of three independent experiments. In panel A, different letters indicate significant differences among tested conditions according to Tukey’s test with a value of *p* of 0.05.

The effect of Pi concentration on stationary phase polyP levels was investigated in *L. paraplantarum* CRL 1905. Cells grown in CDM supplemented with different Pi concentrations presented a Pi dependent-polyP accumulation at 48 h (Figure 1A). Those grown in media containing Pi concentrations below 60 mM presented low polyP levels in stationary phase. In contrast, high polymer levels were observed in cells grown in media supplemented with 60 mM Pi. Based on this critical concentration, media containing sufficient (2 mM, CDM-P) and high Pi (60 mM, CDM + P) were selected for subsequent studies. The analysis of polymer content through the growth curve revealed that cells cultivated in both media exhibited high polyP levels during exponential phase (7 h, see in Figure 1B). The achieved elevated polyP levels were maintained in CDM + P cells for up to 120 h, whereas significantly declined in those grown in CDM-P during the stationary phase (Figure 1B). It is worth mentioning that there was no Pi deficiency in any of the tested conditions and that media Pi concentration did not affect cell growth (Figure 1C) nor the final pH, starting in values of 6 and reaching ~4 after 24 h (data not shown).

### 3.2 High Pi concentration in the culture media enhances survival of *Lactiplantibacillus paraplantarum* CRL 1905

**Figure 2:**
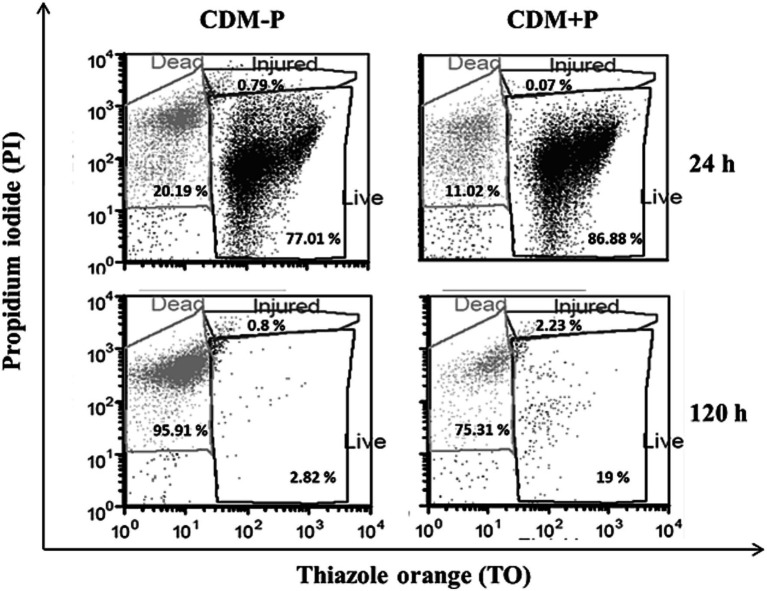
Survival of *Lactiplantibacillus paraplantarum* CRL 1905 grown in high and sufficient Pi conditions analyzed by flow cytometry at 24 and 120 h of culture. According to the cell state, results were grouped into three quadrants in: live, injured or dead. Dot plots are representative of three independent experiments and values are the average of the obtained percentages.

Considering that environmental Pi regulates polyP levels in *L. paraplantarum* CRL 1905, further physiological responses to differential Pi concentrations were studied in the stationary phase. Viability assay showed that cell population in both media was approximately 10^7^ CFU mL^−1^ at 24 h. Subsequently, viable cells in CDM-P started to decrease, reaching to 3 × 10^4^ CFU mL^−1^ at 120 h, whereas cells cultured in CDM + P presented values of ~10^6^ CFU mL^−1^. In addition, a flow cytometry analysis was performed to characterize the cell population in both media (Figure 2). The percentage of living cells at 120 h was higher when grown in CDM + P (~20%) compared to those grown in CDM-P (less than 3%), whereas most of the cells were alive at 24 h in both media (~ 80%). It should be noted that the effects of modifying the Pi concentration were also evident in a complex medium. Indeed, *L. paraplantarum* CRL1905 cells grown in MRS with the addition of Pi to achieve 60 mM maintained high polyP levels and enhanced viability in stationary phase when compared to those grown in MRS (20 mM Pi) (data not shown).

### 3.3 Antioxidant capacity of *Lactiplantibacillus paraplantarum* CRL 1905 increases by high Pi concentration

A combined approach was performed to compare antioxidant ability of cells grown in each condition, including the determination of the cellular scavenging activity on DPPH radicals, the malondialdehyde content (MDA, as a measure of lipid peroxidation), and the tolerance to exogenous hydrogen peroxide. According to results in Table 1, there was a significant decrease in the scavenging capacity of CDM-P cells after 48 h. In contrast, the scavenging ability of CDM + P cells was maintained through time, with values similar to those observed during the exponential growth phase. In addition, CDM-P cells exhibited an enhanced MDA production compared to cells grown in high Pi, indicating stronger damage at the lipid level in the first condition. Furthermore, after 15 min-treatment with H_2_O_2_, cells grown in sufficient Pi medium were more affected than that of CDM + P cells, producing a drop in viability of approximately one order of magnitude (Table 1). It is noteworthy that the scavenging activity, MDA content and exogenous peroxide tolerance during the exponential growth phase revealed no significant differences between both conditions (Table 1). Together, results suggest that CRL 1905 cells grown in high Pi condition exhibited a higher ability to neutralize reactive oxygen species (ROS) during stationary phase, exerting a better antioxidant response.

### 3.4 Morphological changes in *Lactoplantibacillus paraplantarum* CRL 1905 induced by different Pi concentrations

**Figure 3:**
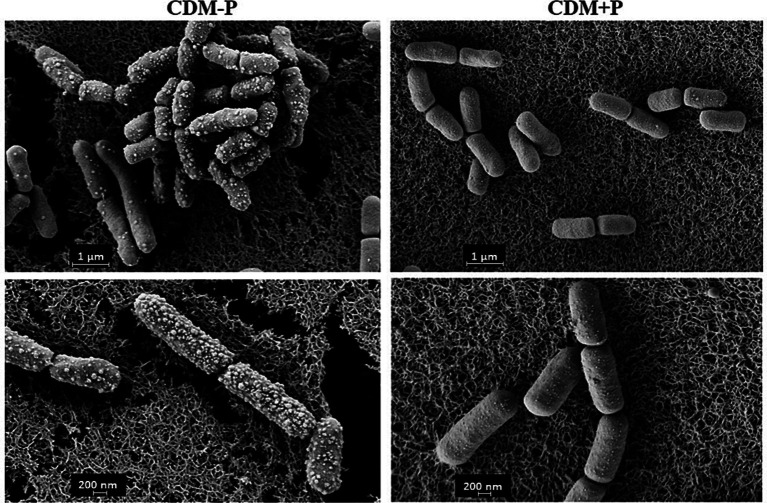
Morphology of *Lactiplantibacillus paraplantarum* CRL 1905 grown in high and sufficient Pi condition. Cells were grown in CDM-P or CDM + P during 48 h and analyzed by SEM. Magnification: 30000x (upper panels) and 50,000x (bottom panels). Data are representative from three independent experiments.

Taking into account the observed differences between stationary phase cells, SEM analysis was carried out to observe possible morphological changes in bacteria grown in both conditions. Figure 3 shows that the 48 h-cells cultured in CDM + P exhibited a typical rod shape and smooth surfaces. However, CDM-P cells tended to clamp and presented rough surfaces due to the appearance of many membrane vesicles (MVs) (Figure 3). It is worth mentioning that the length of cells grown in sufficient Pi medium increased ~25% compared to those grown in high Pi condition.

**Table 1:** *Lactiplantibacillus paraplantarum* CRL 1905 antioxidant capacity grown in different conditions.

		CDM-P	CDM + P
% Scavenging	7 h	24.20 ± 1.32^b^	29.65 ± 0.52^b^
	48 h	10.99 ± 3.10^a^	28.98 ± 3.31^b^
MDA content	7 h	136.48 ± 26.90^a^	141.64 ± 15.48^a^
(pmol mg protein^−1^)	48 h	531.40 ± 42.33^c^	224.53 ± 22.71^b^
% Survival H_2_O_2_	7 h	100	100
	48 h	82.48	93.83

Values of different antioxidant capacity indicators in *Lactiplantibacillus paraplantarum* CRL 1905 grown in CDM-P and CDM + P. Each data represents the mean ± standard deviation from 3 independent experiments. Data within rows followed by the same letter are not significantly different according to Tukey’s HSD (*p* > 0.05).

### 3.5 Impairment of biofilm formation by high Pi concentration in the culture media

**Figure 4:**
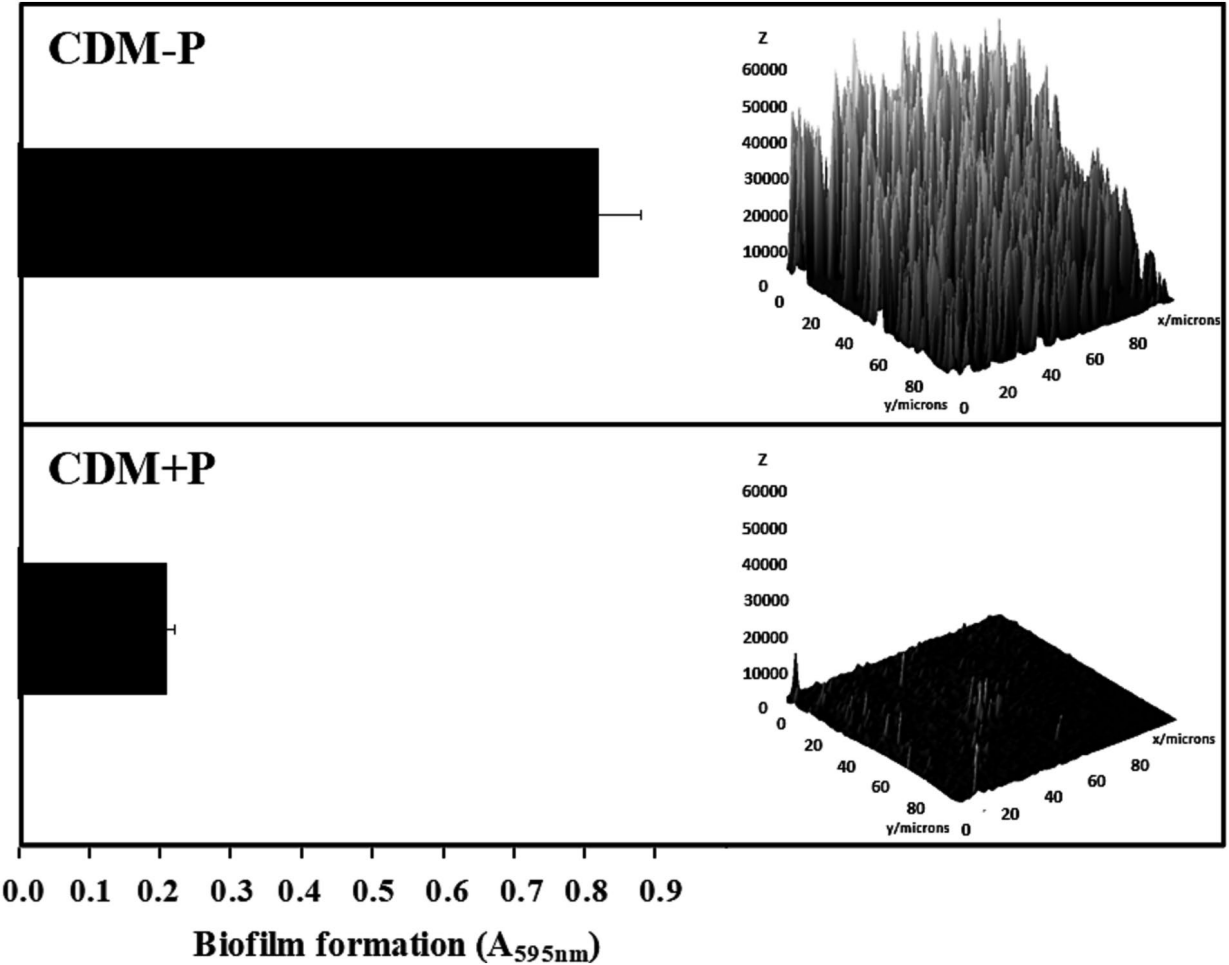
Effect of Pi concentration on the biofilm formation in *Lactiplantibacillus paraplantarum* CRL 1905. Cells were grown in CDM-P and CDM + P during 48 h. Cultures were diluted in fresh medium, containing the respective Pi concentrations, and biofilm formation was quantified after 48 h of incubation by crystal violet (bars graphic) or visualized and analyzed by CLSM (3D graphic constructed with ImageJ software). Data are representative of at least three independent experiments.

The ability of CRL 1905 strain to form biofilm was studied in both conditions at 48 h (Figure 4). Biofilm formation was enhanced when cells were grown in sufficient Pi medium, being approximately 3-fold higher than that produced in high Pi medium (Figure 4, left). As visualized by CLSM, cells grown in CDM-P produced a thicker biofilm than those grown in CDM + P (Figure 4, right), in correlation with the quantitative results obtained through the cristal violet technique.

### 3.6 Media Pi concentration influenced protein expression profile during the stationary phase

To evaluate possible effects of extracellular Pi concentration on *L. paraplantarum* CRL 1905 protein profile during stationary phase, a comparative proteomic analysis was carried out with cells grown in the differential conditions during 48 h. CDM + P *versus* CDM-P expression profile showed 46 differentially expressed proteins (> 1.5 folds, value of *p* < 0.05), from which 28 were up-regulated and 18 were down-regulated. As shown in Table 2, proteins involved in glycolysis, pyruvate metabolism, and pentose phosphate pathway (PdhA, PdhB, PdhC, Pox3, Ack2, PflB, GlpK1, Xfp, RpiA, GalM3, RbsK, and Pgm1), and in nucleotide metabolism (Upp, PyrB, and PyrR) were upregulated under high Pi conditions. On the contrary, chaperones implicated in stress response (Hsp1 and Hsp3) and enzymes involved in the modification of the cell surface (Lp_3421, Acm2, Alr, MurF, and FlmA) were down-regulated in cells grown in CDM + P when compared to those grown in CDM-P (Table 3). According to COG classifications, up-regulated proteins were mainly involved in “carbohydrate transport and metabolism,” “energy production and conversion,” and “nucleotide transport and metabolism,” while most of the down-regulated proteins belonged to the “amino acid transport and metabolism,” “cell wall/membrane/envelope biogenesis,” and “posttranslational modification, protein turnover, chaperones” categories (Tables 2, 3, respectively).

**Table 2:** *Lactiplantibacillus paraplantarum* CRL 1905 up-regulated proteins in CDM + P *versus* CDM-P.

Functional category^a^	Gene	Accession number^b^	Description	Fold change
Amino acid transport and metabolism	*lp_1722*	F9UP80	4-aminobutyrate aminotransferase	*
	*gdh*	F9UMW9	Glutamate dehydrogenase	1.6
Carbohydrate transport and metabolism	*xfp*	Q88S87	Phosphoketolase 2	2.6
	*rpiA*	Q88YY5	Ribose-5-phosphate isomerase	1.9
	*lp_3545*	F9UUK7	D-arabitol-phosphate dehydrogenase	3.9
	*galM3*	F9UUF3	Aldose 1-epimerase	*
	*rbsK*	F9UQH2	Ribokinase	1.7
	*pgm1*	F9UT12	Phosphoglyceratemutase family protein	1.8
Energy production and conversión	*pdhA*	F9UQ93	Pyruvate dehydrogenase complex, E1 component, alpha subunit	*
	*pdhB*	F9UQ92	Pyruvate dehydrogenase complex, E1	2.8
	*pdhC*	F9UQ91	Component, beta subunit Pyruvate dehydrogenase complex,	4.9
	*pox3*	F9URC8	Dihydrolipoamide acetyltransferase	*
	*ack2*	F9UTR4	Pyruvate oxidase	*
	*glpK1*	Q88ZF1	Acetate kinase	1.8
	*pflB*	F9UTJ5	Glycerol kinase 1	*
	*lp_2150*	F9UQ89	Formate C-acetyltransferase Malate/L-lactate/L-2-hydroxyisocapronate dehydrogenase	1.6
Nucleotide transport and metabolism	*upp*	Q9RE01	Uracil phosphoribosyltransferase	*
	*pyrB*	P77883	Aspartate carbamoyltransferase	1.8
	*pyrR*	P71479	Bifunctional protein PyrR 1	1.7
Replication, recombination and repair	*gyrA*	F9US38	DNA gyrase	*
Secondary metabolites biosynthesis, transport, and catabolism	*lp_3666* ^c^	F9ULL3	Aromatic compound hydratase/ decarboxylase	4.9
Translation, ribosomal structure and biogenesis	*frr*	Q88VJ7	Ribosome-recycling factor	*
	*rplW*	Q88XY4	50S ribosomal protein L23	*
	*rpmE2*	Q88Z52	50S ribosomal protein L31	5.7
Function unknown	*lp_2350*	F9UQQ2	Lipoprotein	*
	*lp_2229*	F9UQF3	Metal-dependent hydrolase, beta-lactamase superfamily II	*
	*lp_2260*	F9UQH9	Extracellular protein, DUF336	*
	*lp_0221*	F9UT26	Family 2,5 diketo-D-gluconic acid-like reductase, NADP dependent	1.7

^a^Functional category according to the Cluster of Orthologous Genes (COGs).

^b^Accession number of each protein in uniprot database.

^c^Exceptionally, due to their biological significance, data with a *p* < 0.1 were considered as significantly different when comparing protein abundance from cells grown in CDM + P compared to those grown in CDM-P. In all other cases, data with a *p* < 0.05 were considered as significantly different.

^*^Proteins expression not detected in cells grown in CDM-P, in at least two replicates.

**Table 3:** *Lactiplantibacillus paraplantarum* CRL 1905 down-regulated proteins in CDM + P *versus* CDM-P.

Functional category^a^	Gene	Accession number^b^	Description	Fold change
Amino acid transport and metabolism	*lp_2263*	Q88V24	N-acetyldiaminopimelate deacetylase	*
	*oppF*	F9UN55	Oligopeptide ABC transporter, ATP-binding protein	2
	*lp_0117*	F9USU0	Glutamine amidotransferase, class I	2
Cell wall/ membrane/ envelope biogenesis	*lp_3421*	F9UUA0	Extracellular protein, Ύ-D-glutamate-m-diaminopimelate muropeptidase	2.8
	*acm2*	F9URD9	Cell wall hydrolase/muramidase	1.7
	*alr*	O08445	Alanine racemase	1.7
	*murF*	F9UKZ5	UDP-N-acetylmuramoyl-tripeptide-D-alanyl-D-alanine ligase	*
General function prediction only	*adh*	F9UQP9	Zinc-type alcohol dehydrogenase-like protein	2.2
Intracellular trafficking, secretion and vesicular transport	*secY*	F9UMM5	Protein translocase subunit SecY	*
Posttranslational modification, protein turnover, chaperones	*hsp1* ^c^	F9USV1	Small heat shock protein	2.6
	*hsp3* ^c^	F9UTM5	Small heat shock protein	3.5
Transcription	*rho*	F9UKY8	Transcription termination factor Rho	*
	*flmA*	F9UL63	Transcriptional attenuator, cell envelope-related, LytR family	1.7
Translation, ribosomal structure and biogenesis	*rnj*	F9UQ67	Ribonuclease J	*
	*rplU* ^c^	Q88WN5	50S ribosomal protein L21	4.4
	*tgt*	Q88V05	Queuine tRNA-ribosyltransferase	1.7
Function unknown	*lp_2589*	F9UR98	Polyphosphate: nucleotide phosphotransferase, PPK2 family	*
	*lp_1,146*	F9UMU7	Lipoprotein, pheromone	1.9

^a^Functional category according to the cluster of orthologous genes (COGs).

^b^Accession number of each protein in uniprot database.

^c^Exceptionally, due to their biological significance, data with a *p* < 0.1 were considered as significantly different when comparing protein abundance from cells grown in CDM + P compared to those grown in CDM-P. In all other cases, data with a *p* < 0.05 were considered as significantly different.

^*^Proteins expression not detected in cells grown in CDM + P, in at least two replicates.

## 4 Discussion

A distinctive trait of LAB is their robustness to maintain viable and functional cells during harsh conditions imposed by technological processes or the GIT. The need to enhance LAB survival for different applications has encouraged researchers to find original methods to improve their fitness and to investigate the underlined mechanisms. Several authors have demonstrated that modifications in culture media can alter different metabolic pathways that lead to physiological changes. Indeed, previous reports demonstrated that environmental Pi modulated polyP levels through time affecting different physiological aspects in bacteria (Schurig-Briccio et al., 2009a; Grillo-Puertas et al., 2014, 2018, and, 2021; Correa Deza et al., 2017). Here, it was demonstrated that *L. paraplantarum* CRL 1905 cells grown in high Pi condition maintained elevated polyP levels during stationary phase, improving bacterial survival and antioxidant capacity. On the other hand, in Pi-sufficient medium, polyP was degraded and biofilm and membrane vesicle formation were enhanced.

Stationary phase cells usually experience nutritional stress or starvation due to the nutrient depletion in the culture medium. During this phase, cells may also be subjected to oxidative imbalance, with ROS accumulation and the consequent cellular damage (Alqarzaee et al., 2021). Interestingly, stationary phase-CRL 1905 cells grown in high Pi condition exhibited a significantly higher viability and DPPH scavenging capacity, lower lipid peroxidation and an increased tolerance to exogenous hydrogen peroxide, when compared to those grown in sufficient Pi condition. In agreement, Schurig-Briccio et al. (2008) observed that *Escherichia coli* grown in media containing high Pi concentration experienced a long-term survival, tolerance to H_2_O_2_ and low MDA levels in stationary phase. Also, it has been reported that different *Oenococcus oeni* strains exhibited variable antioxidant abilities that were strain and media-dependent (Su et al., 2015). It is worth to mention that *Lactobacillus* spp. usually exhibit antioxidant potential to mitigate oxidative stress in host cells and tissues, a feature closely linked to their health-promoting properties (Tang et al., 2017; Rwubuzizi et al., 2023). Present results indicate enhanced viability and a stronger antioxidant defense capacity of CDM + P cells of CRL 1905, increasing their robustness to be better adapted for harsh conditions and self-protection against oxidative damage. This potent antioxidant activity represents a promising tool for promoting health benefits.

Bacterial cells undergoing adverse conditions can exhibit changes in morphology, including cellular aggregation, elongation, and alterations in the cell surface (Ingham et al., 2008; Devi and Anu-Appaiah, 2018). Here, notable changes in cell morphology and behavior in response to different growth conditions were observed. Cells grown in CDM-P increased their length, produced membrane vesicles, and improved their capacity to form biofilm compared with those grown in CDM + P. Similar results were observed by Romano et al. (2015), who demonstrated that *Pseudovibrio* sp. FO-BEG1 underwent a prominent cellular elongation under Pi limitation, as a way to increase surface area and enhance nutrient uptake, to cope with the imposed stress condition. The production of MVs in bacteria is also related to stress response processes, such as delivery of virulence factors, quorum sensing, biofilm development and immunomodulation (Pathirana and Kaparakis-Liaskos, 2016; Toyofuku et al., 2017; Dean et al., 2019). Studies carried out on *Pseudomonas putida* demonstrated that cells grown under stressful conditions released MV as a mechanism to alleviate it (Heredia et al., 2016). Furthermore, the CRL 1905 biofilm formation ability in the sufficient Pi medium may be related to the appearance of MVs under this condition. Eberlein et al. (2018) proposed that the release of these vesicles led to an increase in cellular surface hydrophobicity and to the formation of cell aggregates and biofilms. The aforementioned phenotypical adaptations in *L. paraplantarum* CRL 1905 grown in sufficient Pi medium would represent mechanisms to cope with the cellular imbalance that take place during late stationary phase. These adaptations seem to be unnecessary when cells grown in CDM + P, condition where they endure stationary phase successfully. The impairment of biofilm formation capacity in *L. paraplantarum* CRL 1905, when grown in CDM + P, may be linked to the maintenance of high polyP levels in late stationary phase, as demonstrated by previous studies, where degradation of preformed polyP was required to form biofilm in several *E. coli* strains, *Gluconacetobacter diazotrophicus*, and *Herbaspirillum seropedicae* (Grillo-Puertas et al., 2012, 2018, 2021).

Proteomic analysis is a useful approach to gain insight on differential metabolic states. The increased expression of proteins involved in glycolysis, pyruvate metabolism, and pentose phosphate pathway, as well as nucleotide metabolism, suggested that cells grown in CDM + P shifted their metabolism toward energy production and biosynthesis, which may enhance long-term cellular survival. It has been reported that the Pi dependent-modulation of polyP levels is associated with the regulation of primary metabolism and general fitness in prokaryotic cells (Schurig-Briccio et al., 2009a). Other authors observed a higher survival after the up-regulation of enzymes involved in the reduction of pyruvate (i.e., PflB, PdhC, AckA, Ldh, among others) under certain stress conditions in *Enterococcus faecalis*, *Bifidobacterium longum*, *L. reuteri*, and *L. rhamnosus* (Koponen et al., 2012; Kaur et al., 2017). The observed up-regulation of pyruvate oxidase enzyme in CDM + P cells may be related to the improved antioxidant activity exerted by CRL 1905 in this condition. Indeed, Papadimitriou et al. (2016) demonstrated that this enzyme was linked to oxidative stress resistance in *L. plantarum* Lp80.

Under stress conditions, cells employ different chaperones to sequester unfolding proteins, reducing the amount of aggregation-sensitive folding intermediates and preventing the accumulation of protein aggregates (Dahl et al., 2015). Here, two chaperone proteins were repressed in cells grown in CDM + P at 48 h, when compared to those grown in CDM-P. Previous reports demonstrated that proteins involved in stress response, such as heat shock and other chaperone proteins, were induced when bacteria enter to the late stationary phase (Cohen et al., 2006; Laakso et al., 2011; Grillo-Puertas et al., 2021). It should be noted that polyP can act directly by a protein-stabilizing chaperone activity and by interactions with redox-active metals, or indirectly controlling stress response pathways in bacteria (Gray et al., 2014; Gray and Jakob, 2015). Indeed, a general state of stress has been reported in polyP-deficient *Pseudomonas* sp. B4, resulting in the upregulation of protein-folding chaperones (Varela et al., 2010). Thus, the downregulation in the CDM + P condition of the stress-related components may be due to the chaperone capacity of the polyP molecule that could provide the cytoplasmic protein quality to overcome a stress condition.

The only enzyme related to polyP metabolism differentially expressed under the studied conditions was PPK2, a putative polyP: nucleotide phosphotransferase. This protein acts as a primary polyP phosphatase that transfers Pi moiety to GDP in the stationary phase of growth (Ishige et al., 2002). The abundance of this phosphatase in CDM-P correlates well with the observed low polyP levels. On the contrary, the absence of PPK2 expression in CDM + P cells reinforces the unusual maintenance of polymer level in the stationary phase.

Proteins involved in the modification of the cell surface were more abundant in CDM-P, correlating well with the biofilm formation capacity observed in this condition. Previous studies reported the involvement of the cell-envelope FlmA and the hydrolase Acm2 (a major autolysin) in *L. plantarum* biofilm development (Muscariello et al., 2013; D’Abrosca et al., 2018). Furthermore, many proteins involved in the biogenesis of cell envelope were overexpressed when *O. oeni* cells were exposed to different stress conditions (Margalef-Català et al., 2016).

Results provide new insights to understand the adaptations in several metabolic pathways that CRL 1905 experiments in response to differential Pi conditions. Cells in high Pi exhibit an advantage fitness during stationary phase compared to those in sufficient Pi, enhancing their potential as a microbial cell factory for applications in health promotion or agriculture. Variations in environmental Pi could constitute a simple strategy triggering phenotypical adaptations to improve survival and tolerance to harsh conditions that lactobacilli must thrive. Studies in Pi surplus condition to elucidate regulatory pathways concerning the presented differential phenotypes in *L. paraplantarum* become of relevance, since there are scarce reports in LAB regarding to the Pi metabolism and the physiological responses to Pi (Alvarez-Martin et al., 2012; Monedero et al., 2017; Alcántara et al., 2023), focused in Pi deficiency. Further studies should be performed to elucidate the molecular regulatory processes that modify the protein profile and generate the pleiotropic responses presented herein.

## Data Availability Statement

The datasets presented in this study can be found in online repositories. The names of the repository/repositories and accession number(s) can be found in the article/supplementary material.

## Author Contributions

MA: Data curation, Formal analysis, Investigation, Methodology, Writing – original draft, Writing – review & editing. MG-P: Formal analysis, Investigation, Supervision, Writing – review & editing. AM: Data curation, Methodology, Writing – review & editing. EH: Formal analysis, Supervision, Writing – review & editing. JV: Conceptualization, Formal analysis, Funding acquisition, Resources, Supervision, Writing – original draft, Writing – review & editing. VR: Conceptualization, Formal analysis, Funding acquisition, Resources, Supervision, Writing – original draft, Writing – review & editing.
